# Emergence of Oncogenic‐Enhancing Hepatitis B Virus X Gene Mutants in Patients Receiving Suboptimal Entecavir Treatment

**DOI:** 10.1002/hep.30423

**Published:** 2019-02-14

**Authors:** Chih‐Lang Lin, Yu‐De Chu, Chau‐Ting Yeh

**Affiliations:** ^1^ Liver Research Unit Keelung Chang Gung Memorial Hospital Keelung Taiwan; ^2^ Community Medicine Research Center Keelung Chang Gung Memorial Hospital Keelung Taiwan; ^3^ Molecular Medicine Research Center Chang Gung University Taoyuan Taiwan; ^4^ Liver Research Center Linkou Chang Gung Memorial Hospital Taoyuan Taiwan

**Abbreviations**
HBV: hepatitis B virus
HCC: hepatocellular carcinoma
HBx: HBV X protein

Entecavir is a widely used nucleoside analogue for antiviral therapy against chronic hepatitis B virus (HBV) infection. Despite its remarkable efficacy in suppressing HBV replication, a substantial proportion of patients with cirrhosis still developed hepatocellular carcinoma (HCC) after entecavir treatment.^1^ Presumably, it is largely attributable to the existing precancerous hepatocytes, which progress into cancer despite effective viral suppression. HBV X protein (HBx) is a well‐known oncogenic protein. Here, we explored an alternative possibility that oncogenic‐enhancing mutations developed in HBx in patients with HCC having received entecavir treatment.

## Case Series

**Figure 1:**
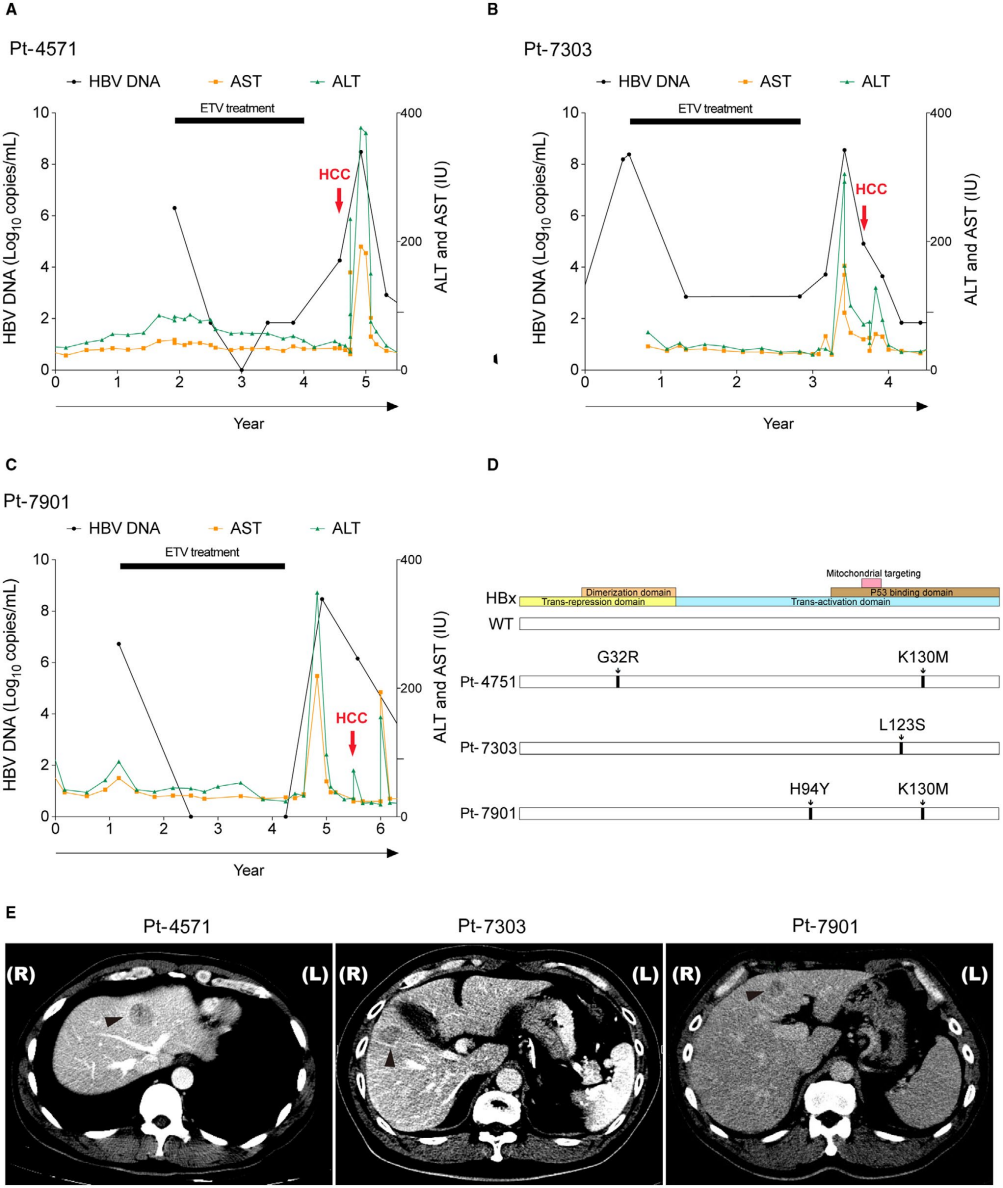
Clinical courses of 3 entecavir‐experienced patients with HCC. (A‐C) The clinical courses of Pt‐4571, a 44‐year‐old male (A), Pt‐7303, a 61‐year‐old male (B), and Pt‐7901, a 54‐year‐old male (C), were depicted. They were all negative for hepatitis B e antigen. At the time of HCC diagnosis, HBV‐DNA was 4.3, 4.8, and 6.2 log_10_/mL, and alpha‐fetoprotein was 51.3, 7.9, and 8.5 ng/mL, respectively. Black circles, HBV‐DNA; orange squares, aspartate aminotransferase; green triangles, alanine aminotransferase; red arrows, time of HCC diagnosis; horizonal bars, entecavir treatment period; horizontal axis, years of follow‐ups. These patients did not receive prior antiviral treatments. All patients were negative for anti‐HCV antibody, not alcoholic, not diabetics, and without family history of HCC. All had liver cirrhosis due to chronic hepatitis B. After treatments, all 3 patients achieved complete remission with no HCC recurrence up to 5 years of follow‐ups. (D) The positions of amino acid substitution mutations on HBx open reading frames. (E) Dynamic computed tomography for the 3 patients with HCC showing wash‐out of contrast medium. All three tumors were hyperdense in arterial phase but became hypodense in early venous phase (Pt‐4571 and Pt‐7303) or in delayed phase (Pt‐7901). All three HCCs were histologically proved. Abbreviations: ALT, alanine aminotransferase; AST, aspartate aminotransferase; ETV, entecavir.

From January 2010 to December 2012, 5 patients previously treated with entecavir developed HCC. HBV‐DNA was detectable at the time of HCC diagnosis in 3 patients (Pt‐4571, Pt‐7303, and Pt‐7901). Entecavir treatment (0.5 mg per day) had been persisted for 2, 2, and 3 years, respectively. HBV‐DNA was incompletely suppressed in Pt‐4571 and Pt‐7303 (< and >2 log_10_/mL, respectively) during the treatment, but completely suppressed in Pt‐7901. Entecavir was withdrawn for all 3 patients according to local insurance policy. HCC was diagnosed 9, 11, and 15 months, respectively, after antiviral withdrawal (Fig. 1A‐C). Serum samples obtained at the time of HCC diagnosis were sent for DNA sequencing. It was found that all 3 patients harbored nonsynonymous mutations in HBx (Fig. 1D), including two liver disease–associating mutations, K130M and H94Y,^2^ and two uncharacterized mutations, G32R and L123S.

**Figure 2:**
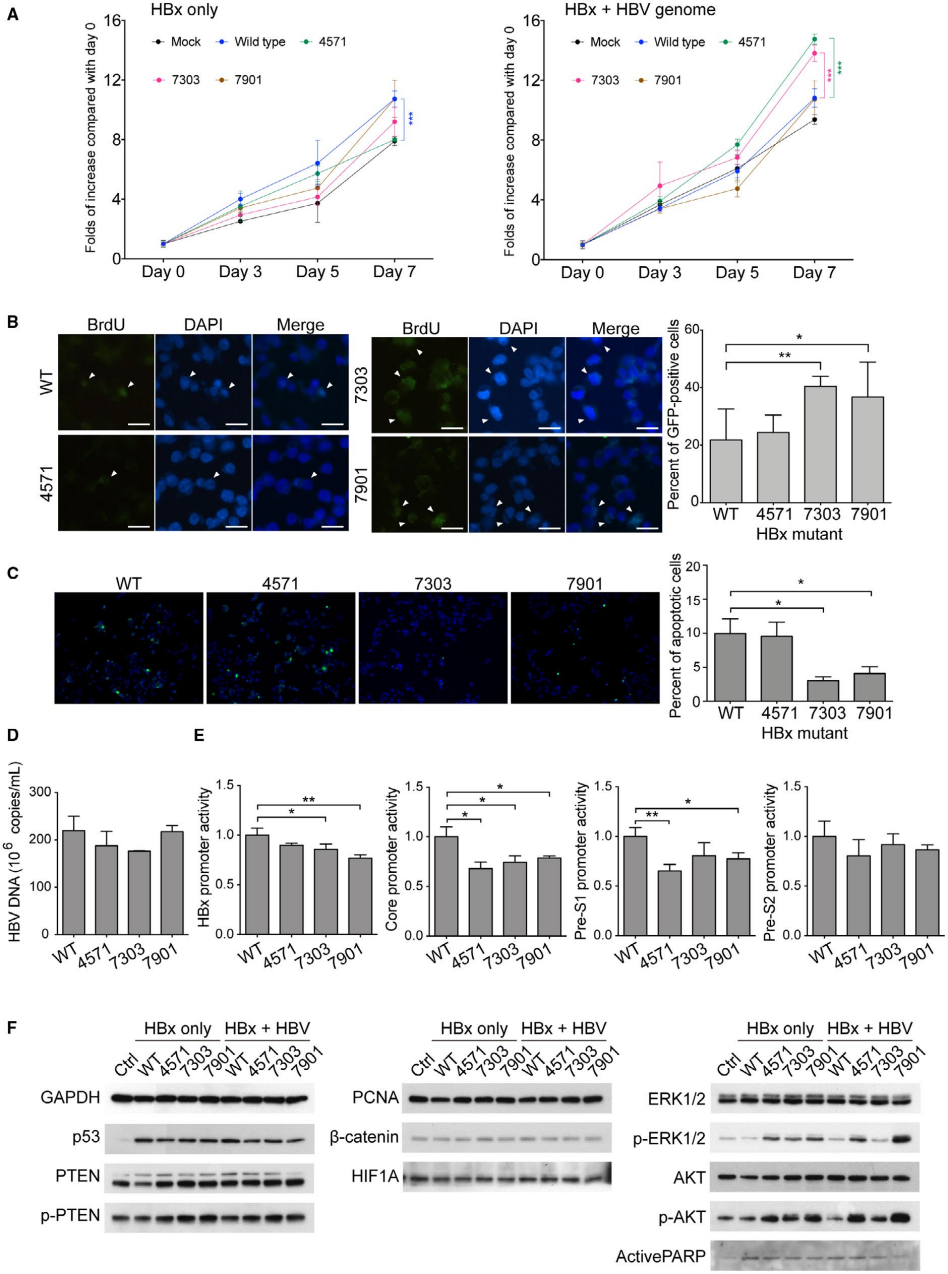
Antiviral treatment‐related HBx mutants had greater cell proliferation‐enhancing and apoptosis‐suppressing abilities, compared with the wild‐type HBx, in the presence of replication‐competent HBV genome. (A) 3‐(4,5‐dimethylthiazol‐2‐yl)‐2,5‐diphenyltetrazolium bromide assays for J7 cells expressing the wild‐type or mutant HBx in the absence (left) or the presence (right) of replication‐competent HBV genome. Black circle, mock control; blue circle, wild‐type HBx; green circle, Pt‐4571‐derived HBx mutant; red circle, Pt‐7303‐derived HBx mutant; brown circle, Pt‐7901‐derived HBx mutant. ***, *P* < 0.001 (pared *t* test). (B) Bromodeoxyuridine incorporation assay in the presence of HBV genome. *, *P* < 0.05; **, *P* < 0.005. (C) Terminal deoxynucleotidyl transferase–mediated deoxyuridine triphosphate nick‐end labeling assays for J7 cells expressing the wild‐type or mutant HBx in the presence of HBV genome. *, *P* < 0.05. (D) HBV‐DNA levels in the mediums of J7 cells expressing the wild‐type or mutant HBx in the presence of HBV genome. (E) The activities of HBx, Core, Pre‐S1 and Pre‐S2 promoters were assayed by luciferase reporter system following transient expression of the wild‐type or mutant HBx. (F) The levels of key molecules in different signaling pathways were examined by western blot. Abbreviations: Ctrl, control; DAPI, 4´,6‐diamidino‐2‐phenylindole; GAPDH, glyceraldehyde 3‐phosphate dehydrogenase; GFP, green fluorescent protein; HIF1A, hypoxia inducible factor 1 alpha subunit; PARP, poly(adenosine diphosphate ribose) polymerase; PTEN, phosphatase and tensin homolog; WT, wild type.

The X coding regions were inserted into the pRc/CMV expressing vector for *in vitro* characterization. When transfected into J7 cells, cell proliferation was significantly enhanced in only the wild‐type‐HBx‐transfected cells. No significant difference was found between the wild type and any of the mutants. However, when cotransfected with a replication‐competent HBV genome, Pt‐7303‐ and Pt‐7901‐derived mutants markedly enhanced J7 cell proliferation, compared with the wild type (Fig. 2A,B). Expression of these two HBx mutants also reduced cell apoptosis (Fig. 2C). All HBx mutants had reduced transactivation ability on core promoter, and each had differentially reduced transactivation abilities on pre‐S1, core, and X promoters (Fig. 2D,E). Finally, western blot analysis showed that phosphorylated‐extracellular signal‐regulated kinase (ERK) and phosphorylated‐protein kinase B (AKT) elevated significantly in HBx mutant–expressing cells (Fig. 2F).

## Discussion

In this report, we discovered that three distinct HBx mutants, harboring various or uncharacterized mutations, emerged after suboptimal entecavir treatment (Fig. 1). Two of them promoted cell proliferation greater than that of the wild‐type HBx, albeit such growth‐enhancing ability was replication‐competent‐HBV‐dependent (Fig. 2). Reviewing the clinical history, it was found that serum HBV‐DNA was elevated in all 3 patients prior to the development of HCC, supporting the requirement of replication‐competent HBV (Fig. 1A‐C). The double mutations identified in Pt‐7901, H94Y and K130M, had been reported in association with severe liver diseases, including HCC.^2^, ^3^

Antiviral resistance has been associated with enhanced hepatocarcinogenesis.^4^ Particularly, the rtA181T mutation developed following lamivudine/adefovir monotherapy is oncogenic, owing to the overlapping S gene mutation (sW172*), resulting in generation of truncated pre‐S/S proteins to trigger oncogenic pathways.^5^ In our cases, however, the polymerase gene did not overlap with the X gene. We speculated that these X mutants were selected in an attempt to overcome antiviral suppression. The transactivation abilities of the X mutants on HBV promoters were altered (Fig. 2E). As such, subcellular dynamic distributions of HBV viral components were altered to compensate for antiviral suppression through yet uncharacterized mechanisms. Supposedly, their transactivation abilities on cellular genes could also be altered, resulting in increased oncogenicity.

In conclusion, we discovered HBx mutations in entecavir‐experienced patients with HCC. The mutants enhanced ERK1/2‐ and AKT‐mediated signaling and promoted cell proliferation in a replication‐competent‐HBV‐dependent manner. According to our findings, suboptimal virological suppression or drug withdrawal can be detrimental to patients with chronic hepatitis B.
