# Supplementary material for: Emergence of Oncogenic‐Enhancing Hepatitis B Virus X Gene Mutants in Patients Receiving Suboptimal Entecavir Treatment
Source: Hepatology. 2019 Feb 14;69(5):2292–6. doi: 10.1002/hep.30423 (PMC6644294; doi:10.1002/hep.30423)
Supplement: Supplementary file 1 [file HEP-69-2292-s001.docx]

**Supplementary Methods**

**Patients**

The study was conducted under approval of institutional review board, Linkou Chang Gung Memorial Hospital, Taiwan. Written informed consent was obtained from all patients included.

**Serological assays and HBV DNA assay**

Serum hepatitis B surface antigen (HBsAg) and hepatitis B e antigen (HBeAg) were assayed by commercially available enzyme-linked immunoassay kits (Roche Diagnostics Corp, Indianapolis, IN, or AxSYM, Abbott Laboratories, Abbott Park, IL; Sanofi Diagnostics, Pasteur, France, or AxSYM, Abbott Laboratories). Serum HBV-DNA levels were quantified by using Roche COBAS TagMan HBV test (Roche Diagnostics, Basel, Switzerland) with a dynamic range between 20 and 1.70 × 10^8^ IU/ml. In this test, 5.82 copies/mL were equal to 1 IU/mL.

**HBV-DNA extraction**

HBV-DNA was extracted as described previously (1). Briefly, 100 μL of serum from each of these 3 patients was mixed with 300 μL digestion buffer (13.3 mM Tris-HCl, pH 8.0; 6.7 mM EDTA; 0.67% SDS; 133 μg/mL proteinase K) and incubated at 55°C for 4 hours. Following twice phenol-chloroform extractions, the HBV-DNA was then precipitated with cold ethanol. The precipitate was dissolved in a final volume of 20 μL TE buffer (10 mM Tris-HCl, pH 8.0; 1 mM EDTA).

**PCR-based amplification of hepatitis B virus X gene for sequencing**

The procedures of PCR were performed as described previously (2). The primer sequences used for HBx gene amplification (for open reading frame) were 5'-GCTCGCAGCCGGTCTGGAGC-3' and 5'-CTTTATAAGGGACAATGTCC-3'. The PCR products were gel-purified and sequence-analyzed.

**Plasmid construction**

The pCMV-HBV plasmid was generated by inserting one copy of a greater-than-unit-length HBV genome (3.37 kb, nt 1820–2990, GenBank accession number X02763) into the pRc/CMV vector (Invitrogen, San Diego, CA) (3). The wild type and mutant HBx gene were PCR amplified by using the primers sequence as mentioned above with further addition of *Kpn*I and *Not*I restriction enzyme sites to the 5’ end, respectively. The PCR products were then inserted into pcDNA3.1 vector (Invitrogen) by using *Kpn*I and *Not*I sites to generate pcDNA3-HBV-XW and pcDNA3-HBV-XMu plasmids. These constructs were all sequence-verified and used for HBx expression in cells.

HBV core promoter (PC), pre-S1 promoter (PS1), pre-S2/S promoter (PS2), and X promoter (PX) were amplified using the pCMV-HBV plasmid as a template and inserted into a pGL3 basic plasmid encoding luciferase (Promega, San Luis Obispo, CA USA) to obtain the reporter plasmids, pGL3-HBV-PC, pGL3-HBV-PS1, pGL3-HBV-PS2, pGL3-HBV-PX. The primers used to amplify these promoters were: for PC with forward primer, 5'-GAGCTCAACAGTATCTGAACCTTTACCC-3' and reverse primer, 5'- GCTAGCCTTGGAGGCTTGAACAGTAG-3'; for PS1 with forward primer, 5'- GAGCTCCGGAAACTACTGTTGTTAGACG-3' and reverse primer, 5'- GCTAGCACCTCCCATGCTGTAGA-3'; for PS2 with forward primer, 5'- GAGCTCAGGTGGGAAACTTTACGGGGCT-3' and reverse primer, 5'- GCTAGCGGAGTTCCACTGCACGGCCT-3'; and for PX with forward primer, 5'- GAGCTCACGGACGGAAACTGCACCTGT-3' and reverse primer, 5'- GCTAGCGCAGCACAGCCTAGCAGCCA-3'. After being digested by *Sac*I and *Nhe*I restriction enzyme, the resulting DNA fragment was puriﬁed by agarose gel electrophoresis and used to replace the corresponding *Sac*I to *Nhe*I fragment in pGL3 basic plasmid. All plasmids were sequence-verified after these constructions.

**Cell culture**

A hepatoma cell line, J7, was used in this study. The Dulbecco’s modified Eagle’s medium (DMEM) containing 10% fetal bovine serum was employed to maintain the cells under standard culture condition. To transiently express HBx gene, transfection was performed by using *Trans*IT-LT1 Transfection Reagent (Mirus, MIR2305) as previously described (4).

**Cell proliferation ability detection**

The cell proliferation rate was assessed as previously described with minor modifications (4). Briefly, the alamar blue was used to replace Thiazolyl Blue Tetrazolium Bromide (MTT) for lower cytotoxicity and simplicity.

**BrdU incorporation and staining assay**

J7 cells in 5x10^4^ seeding density were plated on coverslips in a 12-well plate before transfection. Co-transfection of HBV genome and wild type or mutant HBx was conducted by utilizing *Trans*IT-LT1 Transfection Reagent (Mirus, MIR2305), according to the instructions provided by the manufacturer. Following another two days of incubation at 37 °C incubator, the medium was refreshed with one containing 10 µM BrdU and the cells were further incubated at 37°C for 1 hour. Subsequently, the cells were washed and fixed for standardized immunofluorescence assay. To probe the incorporated BrdU, the anti-BrdU antibody (Proteintech, 66241-1-Ig) was used in a 1:100 dilution. The ratios of BrdU-positive cells to DAPI-positive cells were calculated in 10 randomly selected fields at ×200 magnification.

**TUNEL assay**

To address the apoptotic activity, the TUNEL assay was performed as previously described (5). Briefly, 5×10^4^ cells were plated on coverslips in a 12-well plate. After co-transfected with plasmids encoding HBV genome and the wild type or mutated HBx, cells were incubated for another two days before washed and fixed. The DeadEnd™ Fluorometric TUNEL System (Promega, G3250) was applied according to the manufacturer's instructions. The TUNEL-stained coverslips were mounted onto slides with Vectashield Mounting Medium with DAPI (Vector Laboratories, H-1200) and immediately examined under a fluorescence microscope. The ratios of TUNEL-positive apoptotic cells to DAPI-positive cells were calculated in 10 randomly selected fields at ×200 magnification.

**Luciferase assay**

The luciferase reporters, pGL3-HBV-PC, pGL3-HBV-PS1, pGL3-HBV-PS2, pGL3-HBV-PX, and pGL3-basic, were used for comparison of promoter activities. In this set of experiments, the Renilla luciferase reporter, pRL-TK, was used for normalization. The cells were co-transfected with three plasmids, including one of the pcDNA3.1 empty vector, pcDNA3-HBV-XW, or various pcDNA3-HBV-XMu (0.3 μg); pRL-TK (10 ng); and one of the pGL3-HBV-PC, pGL3-HBV-PS1, pGL3-HBV-PS2, pGL3-HBV-PX, or pGL3-basic (0.3 μg). Lipofectamine 2000 was used for transfection according to the manufacturer’s instruction. The cells were harvested after 24 h and lysed in 1× lysis buffer. Luciferase activity was determined using the Dual luciferase reporter assay system (Promega, San Luis Obispo, CA USA) on a luminometer (TD-20/20, Sunnyvale CA, USA) according to the manufacturer’s instruction.

**Western blot and antibodies**

The protein extraction and western blot analysis were conducted as described previously (6). The rabbit antibodies against GAPDH (Proteintech, 10494-1-AP, 1:10000), HBx (abcam, ab39716, 1:2000), p53 (abcam, ab2433, 1:2000), PTEN (Cell Signaling, #9552, 1:5000), phospho-PTEN (Cell Signaling, #9551, 1:2000), PCNA (abcam, ab15497, 1:5000), HIF1A (Santa Cruz, sc-10790, 1:1000), ERK1/2 (Cell Signaling, #4695, 1:5000), phospho-ERK1/2 (Cell Signaling, #4376, 1:2000), AKT (abcam, ab18785, 1:5000), phosphor-AKT (Cell Signaling, #4060, 1:2000) and mouse antibodies against beta-catenin (BD Biosciences, 610154, 1:2000), cleaved-PARP (BD Biosciences, 552597, 1:1000) were used in this study.

**References**

1. Yeh CT, Liang KH, Chang ML, Hsu CW, Chen YC, Lin CL, Lin WR, et al. Phenotypic and Genotypic Shifts in Hepatitis B Virus in Treatment-Naive Patients, Taiwan, 2008–2012. Emerging Infect Dis 2017;23:820-821.

2. Lin CL, Chien RN, Hu CC, Lai MW, Yeh CT. Identification of hepatitis B virus rtS117F substitution as a compensatory mutation for rtM204I during lamivudine therapy. J Antimicrob Chemother 2012;67:39-48.

3. Yeh CT, Shen CH, Tai DI, Chu CM, Liaw YF. Identification and characterization of a prevalent hepatitis B virus X protein mutant in Taiwanese patients with hepatocellular carcinoma. Oncogene 2000;19:5213-5220.

4. Huang YH, Tseng YH, Lin WR, Hung G, Chen TC, Wang T-H, Lee WC, et al. HBV polymerase overexpression due to large core gene deletion enhances hepatoma cell growth by binding inhibition of microRNA-100. Oncotarget 2016;7:9448-9461.

5. Lai MW, Liang KH, Lin WR, Huang YH, Huang SF, Chen TC, Yeh CT. Hepatocarcinogenesis in transgenic mice carrying hepatitis B virus pre-S/S gene with the sW172* mutation. Oncogenesis 2016;5:e273.

6. Chu YD, Wang WC, Chen SA, Hsu YT, Yeh MW, Slack FJ, Chan SP. RACK-1 regulates let-7 microRNA expression and terminal cell differentiation in Caenorhabditis elegans. Cell Cycle 2014;13:1995-2009.
